# Supplementary material for: Causal Relationship between Aging and Anorexia Nervosa: A White-Matter-Microstructure-Mediated Mendelian Randomization Analysis
Source: Biomedicines. 2024 Aug 16;12(8):1874. doi: 10.3390/biomedicines12081874 (PMC11351342; doi:10.3390/biomedicines12081874)
Supplement: Supplementary file 1 [file biomedicines-12-01874-s001.zip › Supplementary Figure/Figure S4/Singe SNP plot of MR analysis for TL to ubm-b-2009.pdf]

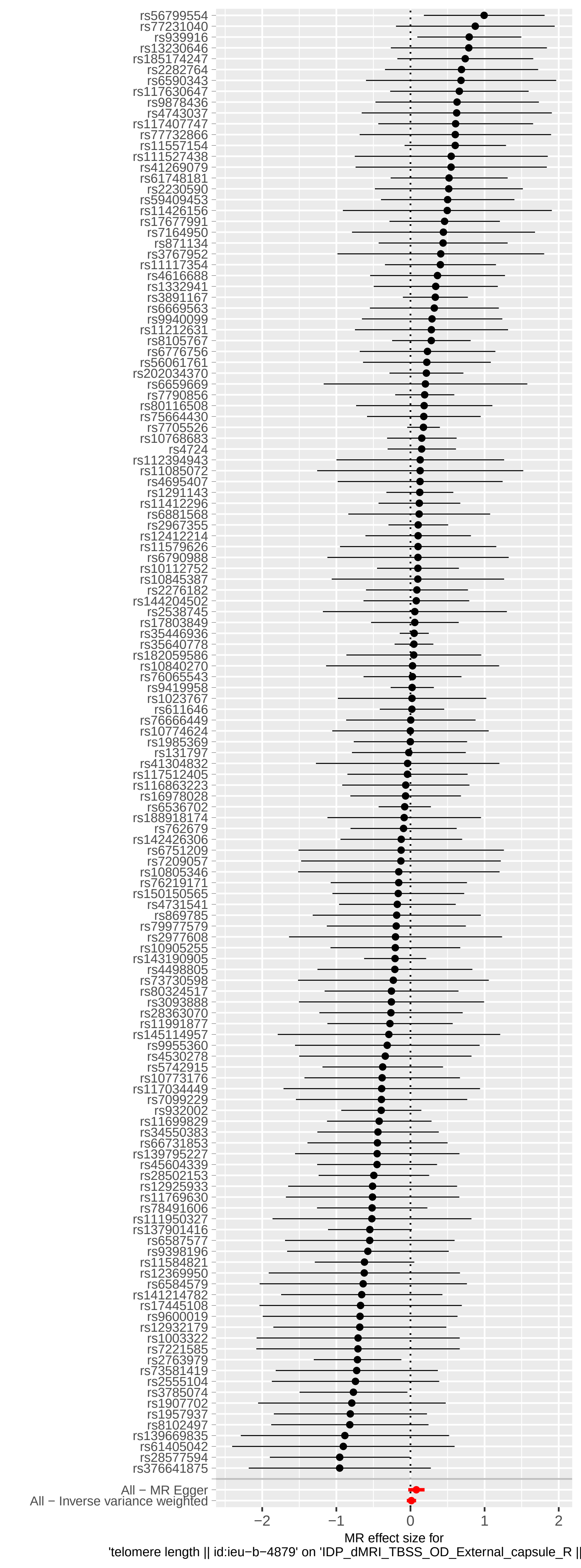
rs56799554
rs77231040
rs939916
rs13230646
rs185174247
rs2282764
rs6590343
rs117630647
rs9878436
rs4743037
rs117407747
rs77732866
rs11557154
rs111527438
rs41269079
rs61748181
rs2230590
rs59409453
rs11426156
rs17677991
rs7164950
rs871134
rs3767952
rs11117354
rs4616688
rs1332941
rs3891167
rs6669563
rs9940099
rs11212631
rs8105767
rs6776756
rs56061761
rs202034370
rs6659669
rs7790856
rs80116508
rs75664430
rs7705526
rs10768683
rs4724
rs112394943
rs11085072
rs4695407
rs1291143
rs11412296
rs6881568
rs2967355
rs12412214
rs11579626
rs6790988
rs10112752
rs10845387
rs2276182
rs144204502
rs2538745
rs17803849
rs35446936
rs35640778
rs182059586
rs10840270
rs76065543
rs9419958
rs1023767
rs611646
rs76666449
rs10774624
rs1985369
rs131797
rs41304832
rs117512405
rs116863223
rs16978028
rs6536702
rs188918174
rs762679
rs142426306
rs6751209
rs7209057
rs10805346
rs76219171
rs150150565
rs4731541
rs869785
rs79977579
rs2977608
rs10905255
rs143190905
rs4498805
rs73730598
rs80324517
rs3093888
rs28363070
rs11991877
rs145114957
rs9955360
rs4530278
rs5742915
rs10773176
rs117034449
rs7099229
rs932002
rs11699829
rs34550383
rs66731853
rs139795227
rs45604339
rs28502153
rs12925933
rs11769630
rs78491606
rs111950327
rs137901416
rs6587577
rs9398196
rs11584821
rs12369950
rs6584579
rs141214782
rs17445108
rs9600019
rs12932179
rs1003322
rs7221585
rs2763979
rs73581419
rs2555104
rs3785074
rs1907702
rs1957937
rs8102497
rs139669835
rs61405042
rs28577594
rs376641875
All − MR Egger
All − Inverse variance weighted
−2
−1
0
1
2
MR effect size for
'telomere length || id:ieu−b−4879' on 'IDP_dMRI_TBSS_OD_External_capsule_R ||
